# The Jewels of Our Genome: The Search for the Genomic Changes Underlying the Evolutionarily Unique Capacities of the Human Brain

**DOI:** 10.1371/journal.pgen.0020080

**Published:** 2006-05-26

**Authors:** James M Sikela

## Abstract

The recent publication of the initial sequence and analysis of the chimp genome allows us, for the first time, to compare our genome with that of our closest living evolutionary relative. With more primate genome sequences being pursued, and with other genome-wide, cross-species comparative techniques emerging, we are entering an era in which we will be able to carry out genomic comparisons of unprecedented scope and detail. These studies should yield a bounty of new insights about the genes and genomic features that are unique to our species as well as those that are unique to other primate lineages, and may begin to causally link some of these to lineage-specific phenotypic characteristics. The most intriguing potential of these new approaches will be in the area of evolutionary neurogenomics and in the possibility that the key human lineage–specific (HLS) genomic changes that underlie the evolution of the human brain will be identified. Such new knowledge should provide fresh insights into neuronal development and higher cognitive function and dysfunction, and may possibly uncover biological mechanisms for information storage, analysis, and retrieval never previously seen.

## Comparative Primate Genomics and the Evolution of the Human Brain

Among the traits that distinguish humans from other primates are a large brain, small canine teeth, bipedalism, an elaborate language, and advanced tool-making capabilities. There are also species-specific changes in skeletal features associated with chewing of food, locomotion, and grasping, and changes related to life span [1]. In addition, humans exhibit reduced hair cover, use sweating more efficiently as a means of thermoregulation [2], and are thought to be more adept long distance runners [3], three human adaptations that may be inter-related. Given that our cognitive abilities, more than anything else, have defined the distinctive evolutionary niche we find ourselves in as a species, it is not surprising that there is a general consensus that it is our brain and its unusual talent for complex thought that is the most significant [4,5]. In contrast, it seems rather remarkable that so little is known about the key genetic events that made our brain unique compared with all other primate and mammalian brains [5,6]. It has been pointed out that a number of neurobiological trends, such as an enlarged neocortex in humans, represent an extension of an evolutionary direction already begun in the brains of other primates that was evident well before the human lineage emerged 5–6 million years ago (Mya). It is estimated that 30–40 Mya neocortical portions of the brain increased in the two emerging anthropoid lineages (platyrrhines and catarrhines) and 8–16 Mya another enlargement occurred in the lineage to the modern hominids [7]. Still, the largest neocortical increase occurred over the past three million years in the human lineage [7], and it is evident that the human brain has abilities, whether in kind or degree or both, that are distinct and unmatched in nature. It should not be surprising then that, for most of us, the genes and genetic changes that are responsible for making the human brain what it is, and for allowing it to do what it uniquely does, have long been among the most prized jewels of our genome.

## Emergence of a Genome-Wide Mindset to Comparative Primate Genomics

A key difference distinguishing our current knowledge of human evolutionary genomics from what it was just a few years ago is one of scope and detail. Instead of a few partial comparative datasets from specific genomic regions, we now have a number of large, genome-wide human and primate datasets on which comparative evolutionary analyses can be based, with more primate genomes to come. While some initial progress has been made in identifying genes potentially important to the evolution of the human brain (Table 1), these discoveries were largely accomplished prior to availability of primate genome draft sequences. With the most current human genome assembly (Build 35) [8], and with the publication of a draft genome sequence of the common chimpanzee *(Pan troglodytes)* [9], our closest living ancestor along with the bonobo *(Pan paniscus),* we now have the unique opportunity to look back and see what roughly 300,000 generations-worth of evolutionary change has done to our respective genomes. In addition to the three primate genome sequences that are either “finished” (human) or in draft form (chimp and macaque), numerous large-scale, cross-species gene expression studies and genome-wide studies of interspecies copy number variation, structural variation, and inversions have been reported. These comprehensive datasets are providing a wealth of new comparative genomic information that promises to yield a much more detailed view of what gene and genomic differences distinguish our species from other primates, as well as what differences are unique to each primate lineage. Given these rapid advances, it is an opportune time to survey this new knowledge and ask what are some of the currently known genomic differences that are unique to our species and which are likely to be key factors underlying human-specific traits, and particularly human-specific cognitive function.

**Table 1:**
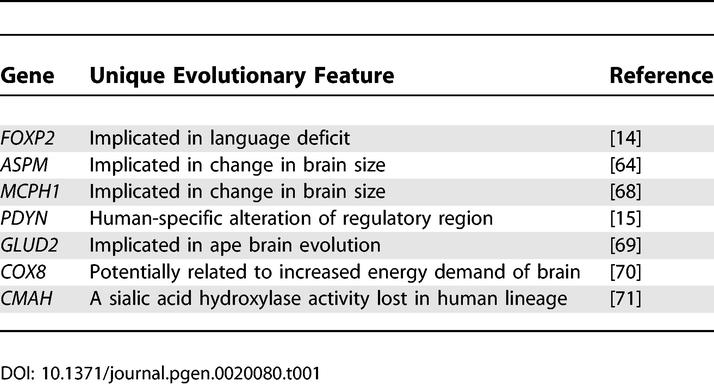
Single-Gene Studies Potentially Related to the Evolution of Human Cognitive Abilities

## Genomic Differences among Primate Lineages

It has been pointed out that the primary molecular mechanisms underlying genome evolution are 1) single nucleotide polymorphisms, 2) gene/segmental duplications, and 3) genome rearrangement [10,11]. In addition, a “less-is-more” hypothesis has been proposed that argues loss of genetic material may also be a source of evolutionary change [12]. Given these factors, what are we learning about their respective roles now that we can compare multiple primate genome sequences?

### 

#### Single nucleotide substitutions.

The initial sequence of the chimp was generated by Whole Genome Shotgun sequencing and covers 94% of the euchromatic portion of the chimp genome, with 3.6-fold coverage for the autosomes and 1.8-fold coverage of the sex chromosomes [9]. Comparison of the relatively finished human genome sequence and draft chimp genome sequence identified 35 million single nucleotide substitutions. After removal of substitutions that show within-species variation, this would translate into a frequency of approximately 1.06%, meaning one could expect to find about one species-specific single nucleotide substitution for every 100 bp of aligned human and chimp genome sequence (this is in contrast to the estimated one single nucleotide substitution for every 1,000 bp when comparing human genomes [13]). The frequency of single nucleotide substitutions may be a slight overestimate due to the fact that the genome of only one chimp (Clint) was sequenced, and some of the predicted interspecies changes may actually be polymorphic in the chimp population. While the great majority of identified changes are likely to be functionally silent, many may have important consequences relevant to protein structure and gene regulation. For example, non-synonymous changes may alter the structure, and potentially the function, of the encoded protein, e.g., FOXP2 [14]. Changes that occur in regulatory regions of a gene may affect the binding site of a transcription factor or other regulator of gene expression, resulting in a change in temporal or spatial expression of a gene, e.g., prodynorphin [15]. Finally, there can be important intronic and coding region single nucleotide substitutions that, while not altering amino acid sequence, can affect exon/intron splicing and, as a result, have significant phenotypic consequences [16].

The presence of unusually high ratios of non-synonymous changes (Ka) to synonymous changes (Ks) in coding region comparisons has been often used as an indicator that positive selection has been at work on one or both of the sequences (Ka/Ks > 1). This approach has been previously applied using human, chimp, and mouse orthologues [17], and recently using the chimp draft sequence, with murid (mouse and rat) sequences as out-groups [9]. Among the functional classes showing the largest number of genes with elevated Ka/Ks ratios were immune function, host defense, apoptosis, spermatogenesis, and chemosensation. In both studies [9,17] neuronal-related genes, such as those encoding neurotransmitter receptors, and synaptic and neurogenesis-related proteins, were not only not among the most positively selected classes but were among those classes at the other extreme (i.e., that showed enhanced constraints on sequence diversity [9]). Another study of human, primate, and mammalian lineages found that Ka/Ks values for neuronal genes have increased in primates (and further on the human lineage within the past five million years) relative to the evolution of neuronal genes in rodents [18].

It is worth pointing out that there are several limitations to using Ka/Ks–based methods to identify evolutionarily important genes. For example, instead of being an indicator of positive selection, elevated Ka/Ks ratios may also be the result of relaxed selection. Conversely, while a minimal number of amino acid changes can result in low Ka/Ks ratios, they can still have major functional effects if they occur at critical locations in a protein.

The publication of the human single nucleotide polymorphism–based HapMap dataset provides another unprecedented new genome-wide resource that not only contains important information about genetic diversity within the human species but also has considerable relevance to human evolution [19]. Six regions of the genome (on Chromosomes 1, 2, 4, 8, 12, and 22) were found to have a paucity of variants and an excess of derived alleles with high frequency in the human population, providing a footprint of the occurrence of selective sweeps [19]. These unusual signatures signify the presence of human genomic changes that, by virtue of being highly adaptive, were rapidly and recently incorporated into the human lineage to the extent that sequences adjacent to the adaptive change have not had time to diverge and have been carried along relatively intact (an example of the so-called “hitchhiking” effect [1]). These six segments will likely be the targets of focused investigations into the search for key human-specific genomic changes.

#### Gene expression differences.

One area that has been actively pursued by multiple groups has been the use of high-density DNA microarrays for genome-wide gene expression studies of multiple primate species using multiple tissues (Table 2; also for a recent review see Preuss et al. [20]). Among the most highly represented functional categories found for genes that consistently show species-specific brain expression changes are transcriptional regulation (e.g., *SMAD1, GTF2I, C21orf33, ZFP36L2*), signal transduction (e.g., *RGL1, PDE4DIP*), lipid metabolism (e.g., *GM2A, SPTLC1*, *PRDX6, OSBPL8*), and cell adhesion (e.g., *COL6A1, THBS4*) [20]. Interestingly, *GTF2I* and *PDE4DIP* also show HLS increases in copy number, as shown by Fortna et al. [34].

**Table 2:**
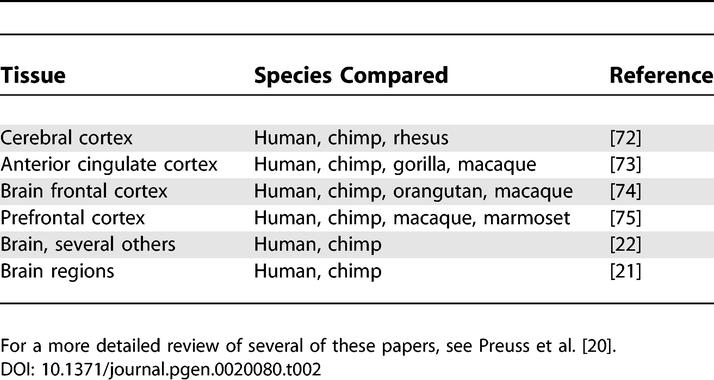
Comparative Brain Gene Expression Studies

As with other expression microarray studies, cross-platform, biological, and experimental variability provide challenges that have to be carefully addressed before meaningful evolutionary changes can be identified. While these efforts have already yielded lists of genes that show interspecies differences in gene expression, deciphering which of these are important to lineage-specific traits still remains a formidable objective. For example, among the factors that can potentially complicate the interpretation of such expression studies are the heterogeneous cellular nature of brain tissue, interindividual variation due to either genetic differences or the many environmental differences that can potentially affect mRNA levels. In addition, in some cross-species experiments (especially using array formats that employ shorter probes, e.g., 20–25 mers) signal differences can be the result of sequence divergence between species rather than a difference in gene expression. While additional steps can be taken to eliminate all microarray data points derived from sequences that differ between human and the other species being compared [21,22], this is only feasible for sequenced species and results in many sequences being removed from the analysis. Finally, while the genes identified by evolutionary comparisons of human and primate brain gene expression may help illuminate important neuronal pathways, such studies have the inherent limitation that, by themselves, they provide little insight regarding the location and nature of the genomic changes underlying the observed expression differences.

#### Frequency and positional biases of structural variations between human and chimpanzee genomes.

Over the past several years a much more detailed view of human genome architecture has emerged and has yielded many valuable and surprising new insights (Table 3) [11,23–30]. For example, it has been well-established that human pericentromeric and subtelomeric regions are particularly dynamic regions [29,31] that are causally related to both disease [32] and evolutionary change [33,34], and harbor a disproportionately high fraction of recent (≤40 Mya) segmental duplications [35] and HLS gene copy number increases [34]. Analysis of the recent chimp sequence indicates that the terminal 10 Mb of hominid chromosomes, encompassing many subtelomeric regions, averages 10% higher sequence divergence than the rest of the genome [9]. In addition, these regions, which comprise approximately 15% of the genome, have elevated local recombination rates, high gene density, and high GC content. Also, if one looks genome-wide, insertions/deletions between chimp and human are abundant, with ~5 million small-to-modest-sized insertions (1 bp to 15 kb) in each species. Remarkably, each genome is estimated to contain 40–45 Mb of species-specific euchromatic sequence [9]. This corresponds to indel differences totaling ~90 Mb of sequence, or 3% of both genomes, and greater than the fraction (1.23%) due to single nucleotide changes. Interestingly the extra human-specific DNA is not randomly distributed but is often found in large segments on a subset of chromosomes, e.g., 1, 9, 13, 16, 19, and Y [9,34,36], with a remarkable 33% (96 of 296) of human duplications being localized in pericentromeric regions [33]. A substantial fraction (70%) of the additional genomic sequences found in chimp also showed a pronounced positional bias, mapping to clusters on Chromosomes 2, 4, and 9 [33]*.* It is noteworthy that the cluster on Chromosome 2 maps to the same site at which two ancestral ape chromosomes fused, telomere-to-telomere, to produce human Chromosome 2 and which contains a striking concentration of human and great ape gene copy number variations [34]. Finally, it is apparent from these studies that both human and chimp have specific genomic locations that serve as sinks for duplicative transposition events, with recently duplicated human sequences being preferentially found at pericentromeric regions and those from chimp (and other African great apes) enriched at subtelomeres.

**Table 3:**
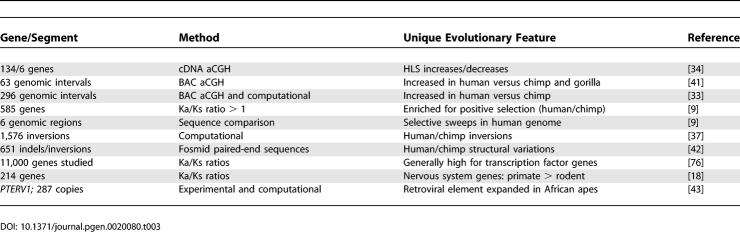
Recent Cross-Species Genome-Wide Sequence and Structural Variation Studies

Sequence inversions between human and chimpanzee are also relatively abundant (>1500), and range in size from 23 bp to 62 Mb [37]. From all of these studies at least two major themes have emerged: 1) structural variations, including copy number differences, indels, and inversions, constitute a significant source of genomic variation between human and chimp, mirroring conclusions obtained by array-based approaches (see below); and 2) to a great extent the degree of genomic difference between human and chimp depends on where in the genome one looks.

#### aCGH and gene and segmental duplication.

Whole Genome Shotgun sequencing was used to generate the chimp draft sequence and is being used for the sequencing of additional primate species. Though it is informative, rapid, and relatively inexpensive, it is also known to have considerable difficulty dealing with highly similar, duplicated sequences (>98%) [26,36]. The most similar duplications are the most problematic to correctly assemble and these will tend to be the most evolutionarily recent. Unfortunately, such recent duplications are also likely to be among the most important to lineage-specific traits found in humans and other primates. Given this limitation of Whole Genome Shotgun sequencing, other genome-wide approaches capable of reliably detecting such recent gene and/or segmental duplications can be expected to fill an important niche both in identifying recent duplications and also in using such information to inform primate genome sequencing centers about potentially problematic regions.

**Figure 1:**
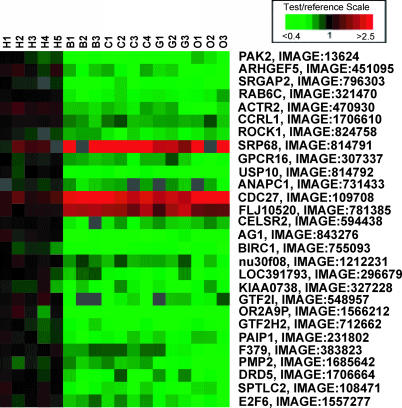
TreeView Image Showing cDNA aCGH Results for Potential Cognition/Brain-Related HLS Genes Brain-related genes listed were obtained from 140 genes predicted by cDNA aCGH to show an HLS change in copy number [34]. H, human; B, bonobo; C, chimpanzee; G, gorilla; O, orangutan.

**Figure 2:**
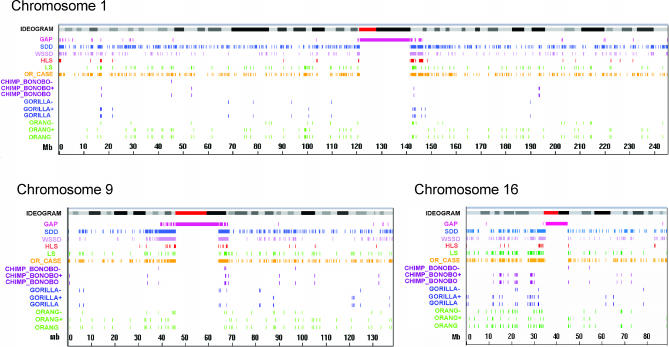
Clustering of Lineage-Specific Gene Copy Number Variations, Segmental Duplications, and Sequence Gaps HLS, LS, and OR_CASE BLAT analysis results were plotted along each chromosome (Build 35) using a modified version of the Genotator annotation browser [77]. HLS and LS refers to those genes identified by Fortna et al. [34] that showed aCGH-predicted gene copy number changes specific for the HLS and for one or more great ape lineages (LS), respectively. OR_CASE refers to those genes for which the aCGH-predicted copy number in human is different from one or more great ape lineages. All available ESTs were downloaded from GenBank for each IMAGE clone in the HLS, LS, and OR_CASE datasets. These ESTs were then aligned to the human genome (Build 35) using a locally installed version of BLAT. All BLAT hits with a score greater than 200 and a percent identity greater than 90% were kept for further analysis. Furthermore, the BLAT hits were parsed down such that only one hit per gene was reported to avoid multiple hits due to isoforms. The LS data set was split into subgroups to indicate orangutan, gorilla, and bonobo plus chimpanzee copy number differences. For these LS subgroups, all differences (gains and losses) are plotted as well as the copy number gains (indicated by a “+”) and copy number losses (indicated by a “−”). Furthermore, the WSSD and SDD annotations [23] were downloaded from UCSC (http://genome.ucsc.edu/) and plotted to illustrate the locations of recent (<40 Mya) segmental duplications in the human genome. Also included is the annotation of the known sequence gaps and an ideogram showing the location of the centromere (red) and the Giemsa staining patterns. Data for Chromosomes 1, 9, and 16 are shown. Data for all chromosomes can be found in Supplementary Figure S1.

The first array-based studies of copy number variants between humans and great apes were carried out comparing limited regions of the genome [38,39]. The first genome-wide (and gene-based) assessment of copy number differences between human and great ape lineages was reported by Fortna et al. [34] and employed array-based comparative genomic hybridization (aCGH) using cDNA arrays [40]. This study identified 1,005 genes that showed lineage-specific copy number variation between human and four great ape species. Of these, 134 and 6 showed HLS increases and decreases, respectively, and many could be linked to possible neuronal functions (Figure 1, Table 4). Striking positional biases were found for these sequences, with the largest clusters being localized near the pericentromeric C-bands of Chromosomes 1 and 9 (and to a lesser degree, Chromosome 16) which are enriched for recent (<40 Mya) segmental duplications and remaining sequence gaps (Figure 2, Figure S1).

More recently, two more genome-wide surveys of interhominoid copy number variation have appeared, using either computational analyses or BAC-based aCGH [33,41]. Data from these three genome-wide array-based studies shows quite strong agreement across platforms. For example, a significant majority (78%) of the gene copy number differences between chimp and human identified by cDNA aCGH [34] were also found by Cheng et al. using a combination of computational and experimental approaches including BAC aCGH [33]. Likewise, of 63 human copy number gains (relative to chimp and gorilla) reported by Wilson et al. [41] using BAC aCGH, 30 segments (48%) had genome coordinates that matched those identified by Fortna et al. [34] using cDNA aCGH. While the BAC aCGH studies only compared human and chimp, or human, chimp and gorilla, the cDNA aCGH report used human and four great ape lineages and, as a result, provides more confidence with regard to predictions of true “lineage-specific” changes (see below). Finally, all three studies gave generally similar results regarding which genomic regions were enriched for recent interspecies copy number variants, e.g., the pericentromeric regions of Chromosomes 1 and 9 known to be expanded specifically in humans. Given that gene duplication is a key engine of evolutionary change, these regions are excellent candidates to harbor genes and/or genomic segments that underlie human-specific traits.

Recently other genome-wide methods have been applied to the detection of structural variations [42] and inversions [37] between human and chimp genomes. While both of these approaches have uncovered a large number of changes (Table 3), the limited use of out-group comparisons affects their ability to confidently identify those changes that are human or chimp lineage-specific.

**Table 4:**
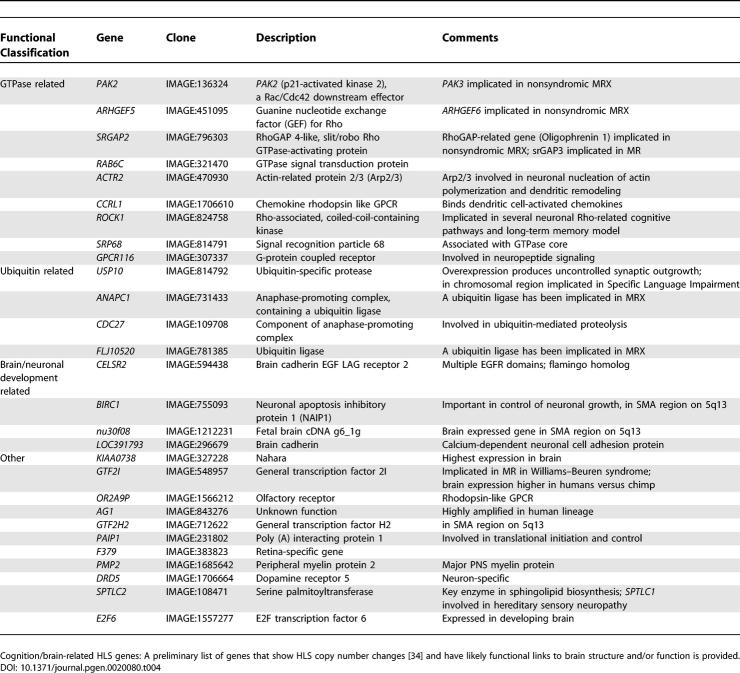
Brain/Neuronal-Related HLS Genes

## Future Comparative Genomic Resources and Directions 

### 

#### More primate genomes and out-groups.

A limitation of the current analysis of the chimp sequence is that, while two murid genomes (mouse and rat) were incorporated in parts of the analysis, comparisons using genome sequences of closely related primate out-groups were not yet feasible. The situation is now changing and, besides the chimp, there are several other primate genomes being sequenced or at least approved for sequencing (http://www.genome.gov/10002154). These include the rhesus macaque (4.6X draft assembly completed and available), orangutan (draft assembly, in process), gorilla (initiated), marmoset (draft assembly, in process), and gibbon (BAC end-sequencing, not started). Finally, a recent commitment has been made to fund the higher-density sequencing of several primate genomes (macaque, marmoset, and orangutan) and to more completely sequence regions of high biological interest in primate genomes (http://www.genome.gov/18016538).

**Figure 3:**
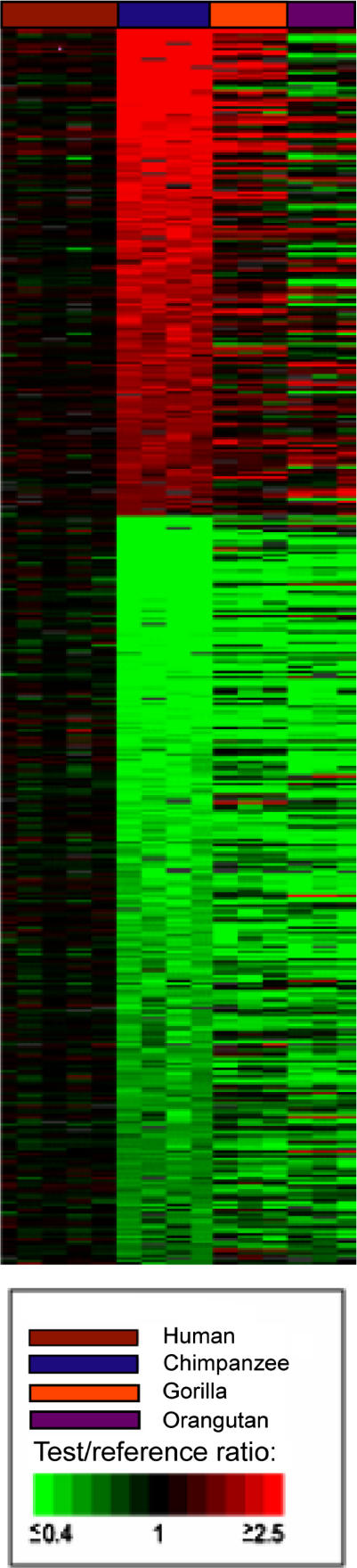
Treeview Image Illustrating the Value of Out-Groups in Identifying Lineage-Specific Copy Number Changes cDNAs shown were selected from a genome-wide dataset [34] to reflect cDNA aCGH–predicted copy number changes between only human and chimp lineages. Also shown are data from gorilla and orangutan for the same cDNAs. Human DNA (labeled green) was used as the reference for all comparisons, while the test samples (labeled red) were human (5), chimp (4), gorilla (3), and orangutan (3). Data illustrate how detection of a copy number difference between human and chimp may not be a reliable predictor that such a change is either human or chimp lineage-specific.

Such studies should provide valuable out-groups for sorting out which changes found between two species are ancestral and which are derived. This can be an important component of comparative genomics, as illustrated, for example, by cDNA aCGH studies across human and four great ape species [34]. In a survey of approximately 30,000 human genes, 353 were identified that showed increased copy number in human compared to chimp. Once other primate out-groups were included, more than half (57% [200/353]) of these were not HLS [34], see for example Figure 3). Interestingly, there were also 47 genes that showed increases or decreases in copy number in three African great ape lineages (bonobo, chimpanzee, and gorilla) compared with human and orangutan [34]. While aCGH does not, by itself, allow one to distinguish whether or not these copy number changes occurred independently in each great ape lineage, for these copy number variants simply comparing human, bonobo, chimp, and gorilla (but not orangutan) would have erroneously suggested that the changes were specific to human. Similarly, it was recently reported that the genomes of the African great apes, but not those of human and orangutan, were targeted, in this case independently, for infection by a specific retroviral sequence 3–4 Mya [43]. From these and other examples, it is clear that the forthcoming primate genomic sequences will help define true “lineage-specific” changes and as a result add considerable value to the already interesting findings obtained so far. Finally, just as having genome sequences available from several different primates will make it possible to more confidently identify HLS genomic changes, the same will be true for each of the individual primate lineages for which genome-wide data will be available. As a result, we can expect to see numerous new discoveries that identify genomic changes specific to each of these primate species. It would therefore seem to be an opportune time to establish programs aimed at sorting out how such changes relate to phenotypic differences among these lineages.

## Approaches to Finding Genes Critical to Human-Specific Cognition 

### 

#### Evolutionary neurogenomics.

Attempts to gain insight into the nature of human cognitive function have traditionally relied on comparative neuroanatomy, which, while helpful, have not yet led to firmly grounded molecular explanations [1,5,44]. Not unexpectedly, the draft of the chimpanzee genome sequence and the imminent availability of other primate sequences is causing this issue to be revisited [45–47]. Attempts at understanding the molecular basis of cognitive function have also been made, but these have largely focused on a few sets of well-known neuronal genes [48–51]. Genes with “unknown” function make up as much as 40% of all human genes [52] but have not been typically incorporated in such models. This bias has been emphasized by Thomas Insel, director of the United States National Institute of Mental Health, who has pointed out that 99% of neuroscience literature focuses on 1% of the genes expressed in the brain [53].

**Figure 4:**
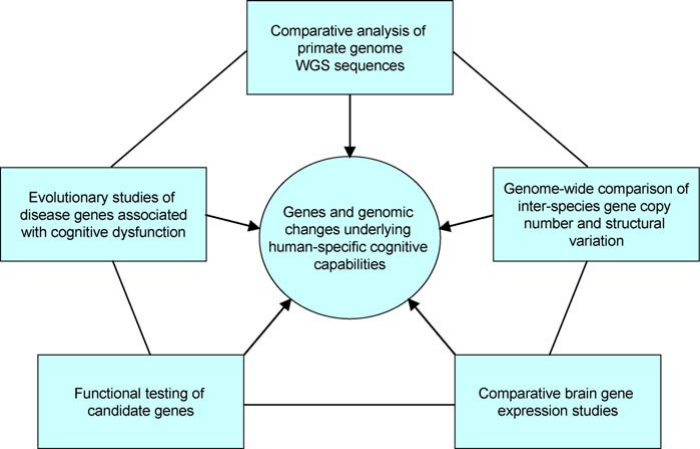
Strategies for Identification of Genes and Genomic Changes Underlying the Evolution of Human-Specific Cognitive Capabilities Listed are various strategies that, either independently or in combination, have the potential to identify gene or genomic changes and pathways relevant to the evolution of human-specific cognitive abilities.

This problem can be aided by the new human and primate genomic resources and strategies that are emerging (Figure 4). Starting from genome-wide datasets and identifying changes that are unique, or more enhanced or reduced, specifically in humans, provides a novel foundation from which to search for genes that are important to human cognitive abilities. Such an approach does not rely on preconceptions about what subset of known genes to focus on, and, as a result, may implicate genes with no known function in such processes, potentially providing new models for how information storage, analysis, and retrieval is accomplished so effectively by the human brain.

What will be the most successful strategy for finding the genomic changes responsible for human-specific cognitive abilities? One of the simplest and most compelling approaches would be to look, at least initially, for extreme genomic changes, such as new gene families or gene or domain hyperamplifications, that are HLS. This rationale has been also expressed as “exceptional duplicated regions underlie exceptional biology” [54]. Such copy number hyperexpansions (>100 copies) have already been reported in chimp compared to human [33], and a gorilla-specific gene amplification has been reported that maps to the subtelomeres of virtually all gorilla chromosomes [34]. Similarly genes have been identified that show extreme HLS amplifications ([34] and unpublished data), and some of these are adjacent to regions, mentioned earlier, that show large, cytogenetically visible, human-specific genomic footprints in certain pericentromeric regions. While these are intriguing candidates, which of these genes (if any) is involved in human-specific cognition remains to be determined.

#### Convergence of studies of cognitive disease and cognitive evolution.

Another strategy that may prove useful is to exploit progress that has been made in identifying genes that underlie diseases of cognition such as mental retardation (MR) [55]. For example, Rho GTPases are thought to be important neuronal signaling molecules and, of seven genes that have been identified that cause MR, three (*PAK3, OPHN1,* and *ARHGEF6*) interact with Rho GTPases [56,57]. Interestingly, among a set of 134 genes that show HLS copy number increases are several that are Rho-related, including *PAK2, SRGAP2, ARHGEF5,* and *ROCK1* [34]. Another example where disease studies may complement evolutionary studies, is in Williams-Beuren syndrome, which is often associated with MR [58]. A recent study implicated the loss of the *GTF2I* gene in the MR of Williams Syndrome [59], and, interestingly, the same gene has a higher copy number specifically in the human lineage [34] and is among those genes showing consistent brain (cortex) gene expression increases (2.5-fold to 4.2-fold) in human compared to chimp [20]. Likewise, regions on Chromosomes 16 and 19 have been implicated in Specific Language Impairment [60], and genes within those regions show elevated copy number in humans [34]. Similarly, linkage hotspots have been identified for other diseases of cognition such as dyslexia [61], and these could be checked to see if these regions co-localize with genes implicated in human evolutionary change. The linking of genes underlying brain diseases to a role in human brain evolution has already proved to be productive. For example, the *FOXP2* gene has been found to underlie a human language deficit and subsequently shown to harbor signs of selection in the human lineage [62]. Also, the gene causing microcephaly in humans (*ASPM*) has undergone rapid evolutionary change in the human lineage and may be related to human's increased brain size [63,64].

#### Functional studies.

Once candidate genes or genomic changes related to human cognition have been found, the next challenge will be how to test them functionally, especially when the function may be largely human-specific. Transgenic approaches using primates are unlikely to be an option for ethical reasons. A more acceptable direction and one that could prove valuable would be to generate transgenic mice using human-specific genes. As mentioned earlier, a substantial amount of the human genome appears to be unique to humans, so there should be numerous candidate genes to test. Such transgenics could also be crossed to see the effects of multiple human-specific genes, and there are numerous behavioral and cognitive tests that are well-established in murine research that could be employed. The same transgenics could also be studied at the molecular and cellular levels to determine what structures, pathways, and processes have been altered, and we could, in so doing, potentially gain insight into what neuronal function(s) may be affected in the human brain. Genes that give encouraging results in transgenic experiments could be used to survey the human population to determine how variations in the genes relate to cognitive deficits or enhancements.

## Concluding Remarks

When Wilson and King published their classic paper [65], the amount of human and chimp sequence upon which they based their conclusions was, by current standards, miniscule. From that early perspective, the unusually high degree of sequence similarity between humans and chimps argued that the considerable anatomical and physiological differences between these species would likely be due to small DNA changes that confer large effects. One of the most important findings to emerge from the latest human and primate genome-wide studies is that a fundamental assumption underlying this model has changed: the interspecies genomic changes are numerous and diverse [9,33,66,67], and, as a result, there appear to be many additional types of genomic mechanisms and features that could also be important to the evolution of lineage-specific traits. Given this new perspective, we now know that the degree of difference between our genome and that of the chimp depends on where, and how comprehensively, we look. The multitude of genomic differences that we now know exists should provide an abundance of fertile genomic ground from which important lineage-specific phenotypes, such as enhanced cognition, could have emerged. 

## Supporting Information

Figure S1 Updated (Build 35) Genomic Locations of Genes Showing Interhominoid Copy Number Changes and Correlation with Recent Segmental Duplications and Sequence Gaps For Chromosomes 1–22, the X chromosome, and the Y chromosome.
See Figure 2 for details.
(162 KB DOC)

## Abbreviations

HLS: human lineage–specific
MR: mental retardation
MRX: X-linked mental retardation
Mya: million years ago
