# Supplementary material for: The Jewels of Our Genome: The Search for the Genomic Changes Underlying the Evolutionarily Unique Capacities of the Human Brain
Source: PLoS Genet. 2006 May 26;2(5):e80. doi: 10.1371/journal.pgen.0020080 (PMC1464830; doi:10.1371/journal.pgen.0020080)
Supplement: Figure S1 — For Chromosomes 1–22, the X chromosome, and the Y chromosome. See Figure 2 for details. (162 KB DOC) [file pgen.0020080.sg001.doc]

Supplemental Figure S1

Updated (Build 35) genomic locations of genes showing interhominoid copy number changes and correlation with recent segmental duplications and sequence gaps. See Figure 2 for details.


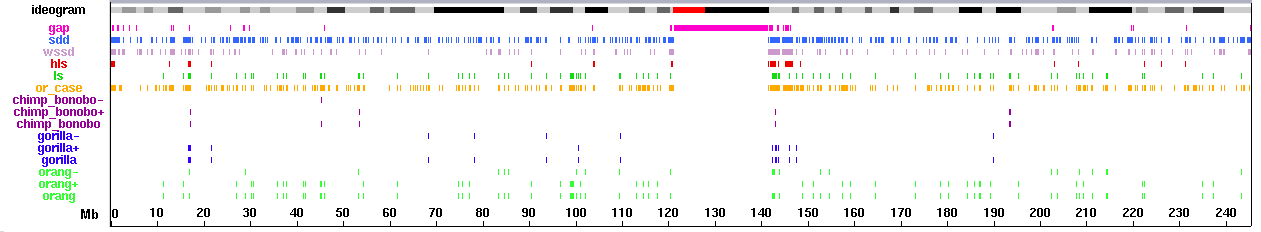
 Chromosome 1


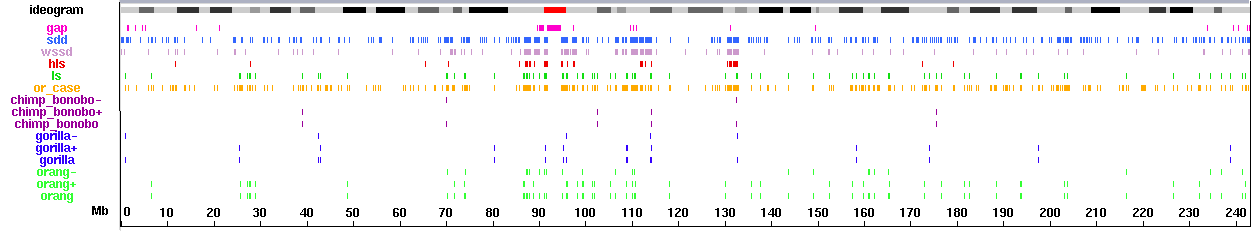
 Chromosome 2

Chromosome 3


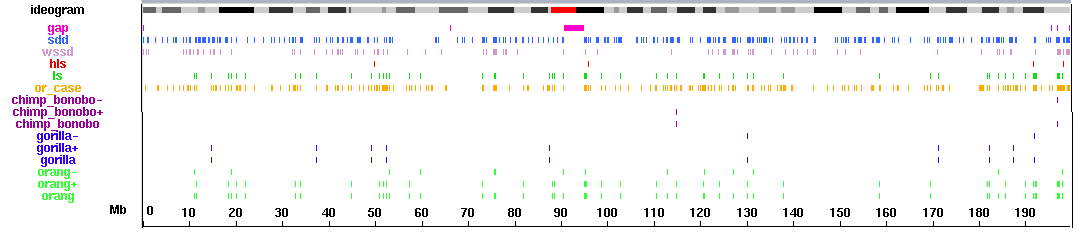


Chromosome 4


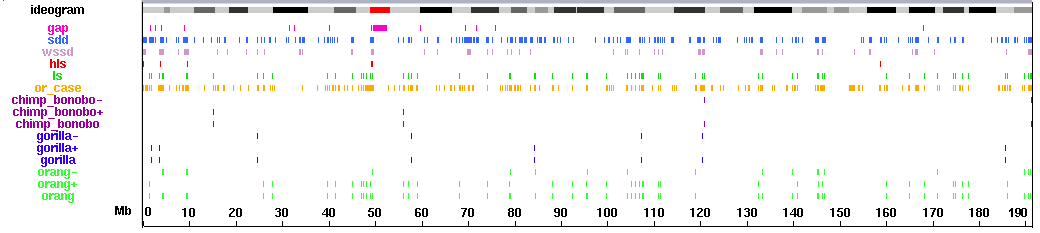


Chromosome 5


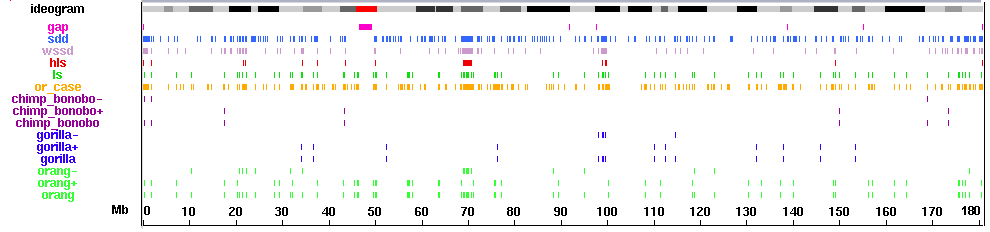


Chromosome 6


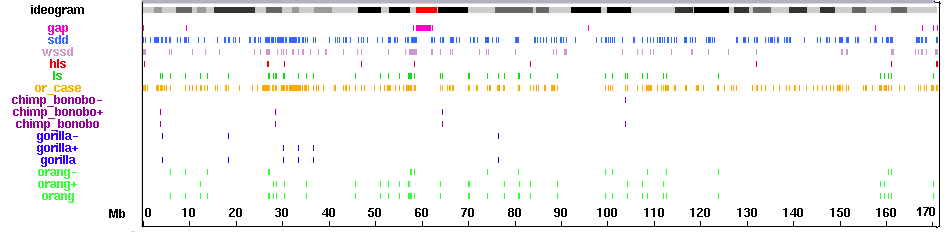


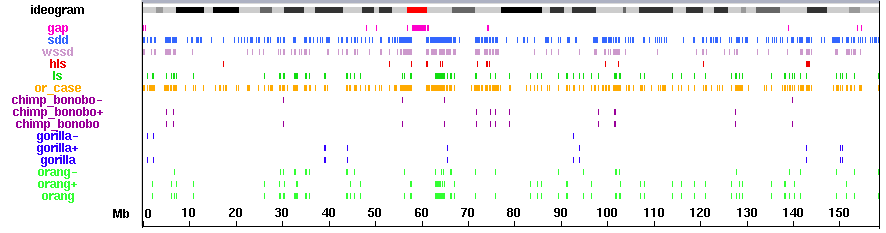

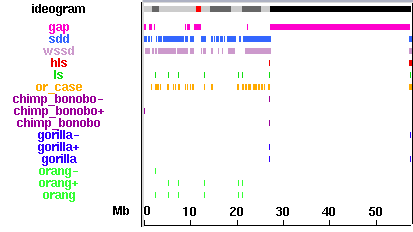
 Chromosome 7 Chromosome Y


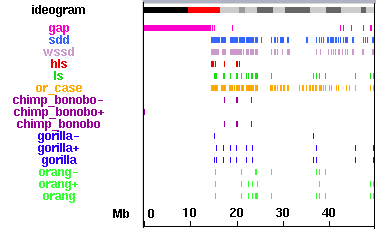
 Chromosome X Chromosome 22


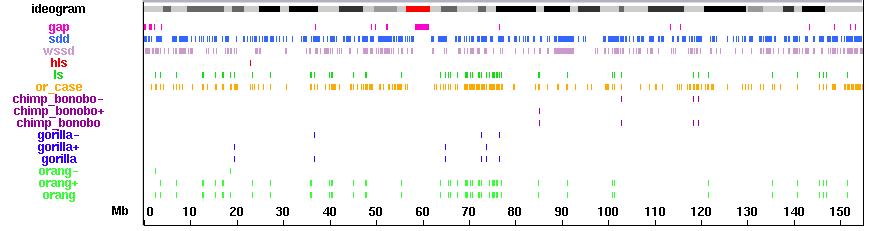


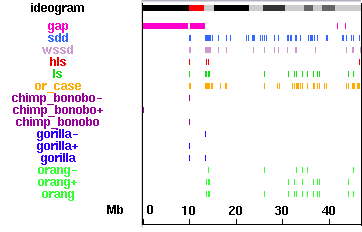
 Chromosome 8 Chromosome 21


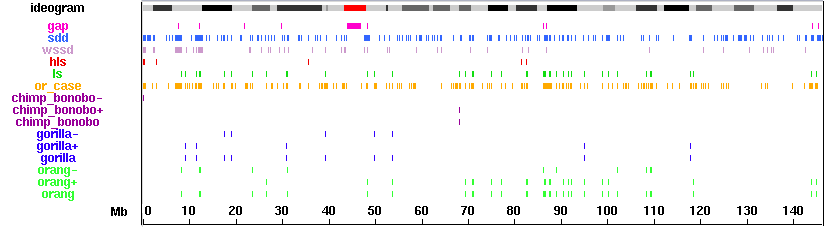


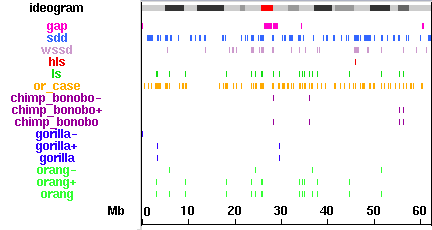
 Chromosome 9 Chromosome 20


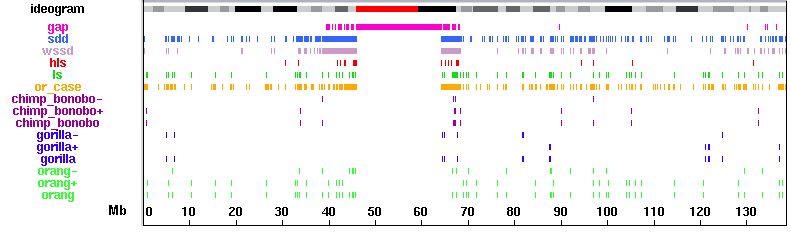


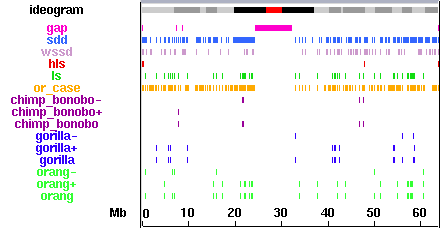
 Chromosome 10 Chromosome 19


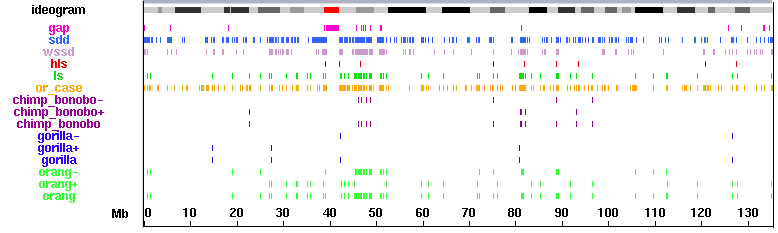


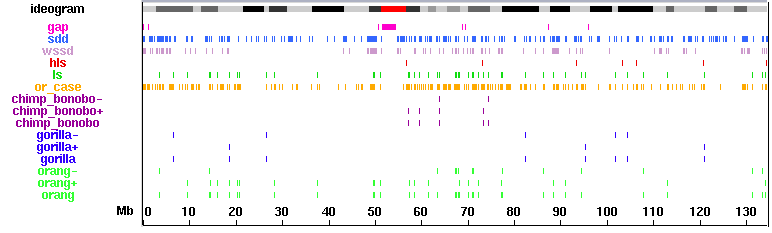

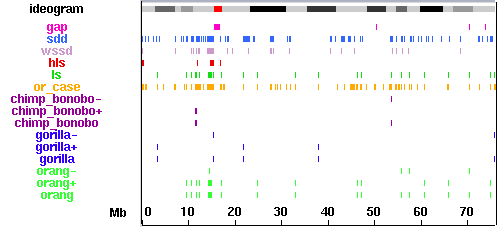
 Chromosome 11 Chromosome 18


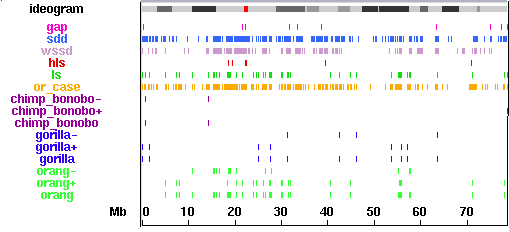
 Chromosome 12 Chromosome 17


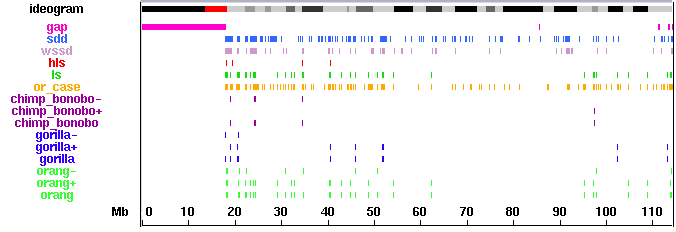

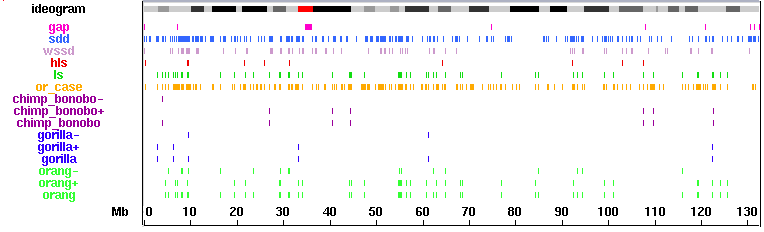


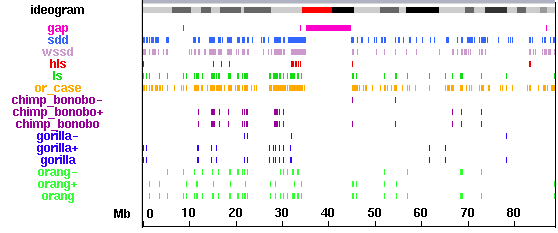
 Chromosome 13 Chromosome 16


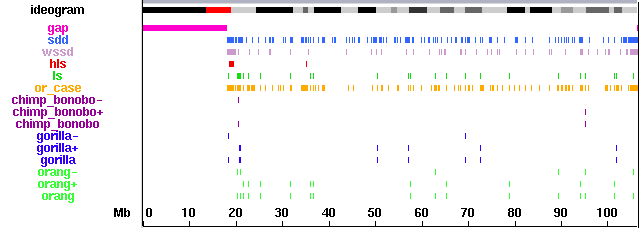

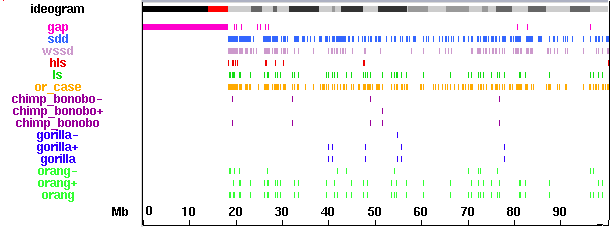
 Chromosome 14 Chromosome 15
